# Prokaryotic Community Structures in a Thermophilic Anaerobic Digestion Reactor Converting Poly(l-Lactic Acid) for a Long Period Revealed by 16S rRNA Gene Amplicon Sequencing

**DOI:** 10.1128/MRA.00679-19

**Published:** 2019-07-18

**Authors:** Takeshi Yamada, Masako Hamada, Misaki Kurobe, Jun Harada, Surya Giri, Hideto Tsuji, Hiroyuki Daimon

**Affiliations:** a Department of Applied Chemistry and Life Science, Toyohashi University of Technology, Toyohashi, Aichi, Japan; b Core for International Relations, Toyohashi University of Technology, Toyohashi, Aichi, Japan; University of Southern California

## Abstract

Little information on poly(l-lactic acid) (PLLA) treatment-associated microbiota in thermophilic anaerobic digestion reactors is available. Here, we provide 16S rRNA gene sequence data on microbiota in a thermophilic anaerobic digestion reactor converting PLLA to methane for 336 days. Data comprising 99,566 total high-quality reads were tabulated at the taxonomic class level.

## ANNOUNCEMENT

Biohydrolysis of poly(l-lactic acid) (PLLA) does not occur at desired levels in anaerobic digestion reactors (1, 2). Therefore, improving chemical hydrolysis by appropriately adjusting weight-average molecular weight (*M*_w_) and crystallinity (*X*_c_) is considered effective for thermophilic anaerobic digestion (2). However, information on PLLA treatment-associated microbiota during thermophilic anaerobic digestion is limited. Here, microbial 16S rRNA gene sequence profiles were analyzed to obtain basic information about prokaryotic communities to achieve efficient PLLA methane fermentation in thermophilic anaerobic digestion reactors.

**FIG 1:**
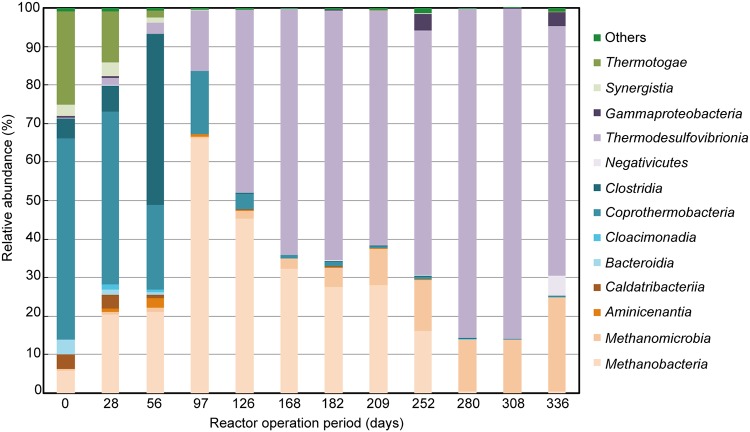
Relative abundance of prokaryotic communities in a thermophilic anaerobic digestion reactor converting poly(l-lactic acid) to methane for 336 days. Each bar represents the class indicated as a percentage of the diversity from the thermophilic anaerobic digestion sludge.

A single anaerobic continuously stirred tank reactor (effective volume, 7.5 liters) was continuously operated for 336 days at an organic loading rate of 0.5 kg · m**^−^**^3^ · day^−1^ and a hydraulic retention time of 50 days. Anaerobic sludge used as the inoculum for this process was collected from a full-scale thermophilic anaerobic digestion reactor (installed in Kitanagoya, Japan). The reactor temperature was maintained at 55°C by a water jacket around the reaction vessel. Chemically hydrolyzable PLLA (adjusted to *M*_w_ = 10,300 and *X*_c_ = 39.9%) was used as the sole substrate. For measurements using gas chromatography described previously (2), methane concentrations in biogas generated from the reactor ranged from 44.4 to 53.5%. Twelve thermophilic anaerobic sludge samples were periodically collected from the reactor (Fig. 1), and DNA was extracted according to the method of Yamada et al. (3). Blend *Taq* polymerase (Toyobo, Osaka, Japan) and a 515F/806R primer set (4) were used to amplify the V4 region of the prokaryotic 16S rRNA gene in each sample. PCR product sequences were determined using the MiSeq platform (Illumina, San Diego, CA, USA) and MiSeq reagent kit v2 (2 × 300 bp) (Illumina) at the Bioengineering Lab. Co., Ltd. (Kanagawa, Japan). Sequence adapter, index, and primer regions were deleted using FASTX-Toolkit v0.0.13 (5). Read sequences of ≤40 bp and low-quality sequences (≤Q20) were discarded using Sickle v1.33 (6). High-quality paired-end reads were merged using PEAR v0.9.10 with default settings (7), discarding reads containing sequences of ≤245 and ≥260 bp with SeqKit v0.8.0 (8). Each operational taxonomic unit (OTU) was assigned to the appropriate taxa using QIIME v1.9.1 (9) and the SILVA database release 132 with 97% identity (10). Relative abundances of representative OTUs (>1%) at the class level for each sample are shown in Fig. 1, and indices used to assess diversity, calculated using QIIME v1.9.1 (9), are summarized in Table 1.

**TABLE 1:** Index for assessing the diversity of microbiota in each thermophilic digester sludge sample collected on different sampling days

Data by reactor operation period (days) (SRA run no.)	Estimated sample coverage	No. of observed OTUs	Analysis measure for:			
			Shannon diversity	Simpson diversity	Chao1 estimator	No. of high-quality reads/sample
0 (DRR180057)	0.99	123	2.61	0.68	184.2	7,469
28 (DRR180058)	0.99	138	2.90	0.75	266.6	7,779
56 (DRR180059)	0.99	93	2.51	0.72	157.7	8,115
97 (DRR180060)	1.00	27	1.43	0.51	45.2	4,754
126 (DRR180061)	1.00	20	1.42	0.57	53.0	4,905
168 (DRR180062)	1.00	12	1.18	0.49	27.0	3,443
182 (DRR180063)	1.00	46	1.40	0.50	67.1	11,688
209 (DRR180064)	1.00	31	1.44	0.54	46.2	9,526
252 (DRR180065)	1.00	90	1.84	0.56	176.7	18,924
280 (DRR180066)	1.00	21	0.75	0.26	33.0	7,082
308 (DRR180067)	1.00	18	0.68	0.25	27.3	8,585
336 (DRR180068)	1.00	37	1.77	0.55	63.3	7,580

High-quality reads of 3,443 to 18,924 bp were obtained from each sample, and relative abundance at the class level was summarized based on 99,566 total high-quality reads from 12 samples. The main prokaryote classes included *Methanobacteria*, *Methanomicrobia*, *Aminicenantia*, *Caldatribacteriia*, *Bacteroidia*, *Cloacimonadia*, *Coprothermobacteria*, *Clostridia*, *Negativicutes*, *Thermodesulfovibrionia*, *Gammaproteobacteria*, *Synergistia*, and *Thermotogae*. Microorganisms converged into the classes *Thermodesulfovibrionia* (15.7 to 85.6%), *Methanomicrobia* (0.5 to 24.6%), and *Methanobacteria* (0.3 to 66.2%) after 97 days of operation (Fig. 1). These data will help identify microbial communities associated with methane production from PLLA in thermophilic anaerobic digestion reactors.

### Data availability.

The 16S rRNA gene amplicon data set has been deposited in the DDBJ Sequence Read Archive (SRA) under the accession number DRP005116 and the SRA run accession numbers DRR180057 to DRR180068.
